# Self‐Esteem and Psychopathology Differentially Relate to Real‐Life and Social Functioning in People With 22q11.2 Deletion Syndrome

**DOI:** 10.1002/jdn.70017

**Published:** 2025-04-24

**Authors:** Tommaso Accinni, Georgios D. Kotzalidis, Emanuele Cerulli Irelli, Massimo Pasquini, Antonino Buzzanca

**Affiliations:** ^1^ Department of Human Neurosciences Sapienza University of Rome, Faculty of Medicine and Dentistry Rome Italy; ^2^ Department of Neurosciences, Mental Health, and Sensory Organs Sapienza University of Rome, Faculty of Medicine and Psychology Rome Italy

**Keywords:** 22q11.2 deletion syndrome, real‐life functioning, self‐esteem

## Abstract

**Background:**

The 22q11.2 deletion syndrome (22q11.2DS) represents a genetic condition at higher risk of transition to psychosis. Both self‐esteem (SE), intended as self‐evaluation based on cognitive and affective elements, and psychotic symptoms may be associated with patients' real‐life functioning. We investigated whether these variables differently correlate with real‐life functioning in 22q11.2DS.

**Methods:**

We recruited 22 patients with 22q11.2DS (DEL, *N* = 22) and 10 with 22q11.2DS and psychosis (DEL‐SCZ, *N* = 10); we administered the Positive And Negative Syndrome Scale (PANSS), the Specific Levels of Functioning scale (SLoF) and the Self Esteem Rating Scale (SERS).

**Results:**

The DEL‐SCZ and DEL groups did not significantly differ on the SERS (*p* = 0.228). The DEL group scored higher than DEL‐SCZ on the SLoF‐total (*p* = 0.006) and on the SLoF‐social functioning (*p* = 0.031). PANSS‐total negatively correlated with SLoF‐total scores (*ρ* = −0.698; *p* < 0.001), with the SLoF‐social functioning (*ρ* = −0.643; *p* < 0.001) and with SERS (*ρ* = −0.391; *p* = 0.036). SERS scores positively correlated with SLoF‐total (*ρ* = 0.545; *p* = 0.003) but not with SLoF‐social functioning.

**Discussion and Conclusions:**

DEL and DEL‐SCZ display similar levels of SE suggesting that this psychological dimension is not associated with psychotic symptoms. Levels of SE and psychopathology differentially relate to real‐life and social functioning in people with 22q11.2DS: Symptom severity is particularly associated with patients' social and interpersonal functioning. Psychological supportive interventions might be useful to improve real‐life functioning in people with 22q11.2DS.

## Graphical abstract

Self‐esteem is not directly associated with psychosis in individuals with the 22q11.2 deletion syndrome (22q11.2DS). Self‐esteem and psychopathology differentially relate to real‐life functioning in individuals with 22q11.2DS: Although the former appears to be associated with total levels of functioning, only the latter is correlated with patients' social functioning.

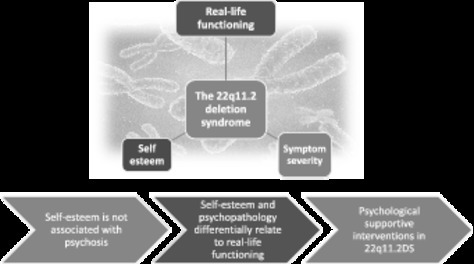

## 1 Background

The 22q11.2 deletion syndrome (22q11.2DS) derives from a dominant microdeletion at the 11.2 strand on the long arm (q) of chromosome 22, representing the most common among known rare copy number variations (CNVs) (McDonald‐McGinn et al. 2015). It is rare, with an incidence of 1:3000 to 1:6000 of new births (Olsen et al. 2018). It involves about 40 coding genes leading to wide phenotypic expressions (Óskarsdóttir et al. 2023; Zamariolli et al. 2023) and neuropsychiatric features; in particular, psychotic disorders are prominent clinical conditions. People with 22q11.2DS show an increased lifetime risk of developing a psychotic illness, with rates ranging from 23% to 43% across studies (Fiksinski et al. 2023; Provenzani et al. 2022; Schneider et al. 2014). Psychotic disorders in 22q11.2DS clinically overlap with those without clear genetic causes (Bassett et al. 2005). For this reason, 22q11.2DS represents a reliable model to study neurobiological proneness and vulnerability to psychosis.

People with schizophrenia suffer from significant impairment of their independent living, without achieving an expected level of autonomy concerning productive activities and social abilities (Galderisi et al. 2014). Integrating psychopathological variables with everyday life skills, cognitive dysfunction, functional capacity, personal resources, perceived stigma and real‐life functioning in such people is critical for improving their general functioning and ensuring effective therapeutic interventions (Galderisi et al. 2018).

Self‐esteem (SE) refers to self‐concept involving both cognitive elements of self‐evaluations and affective elements relating to how one feels because of these evaluations (Wells 1976). Low SE is strongly associated with affective conditions (Silverstone and Salsali 2003), but also with schizophrenia (Rossi et al. 2017), hindering recovery and general functioning (Hofer et al. 2023). Lower SE levels were found in clinical high‐risk for psychosis individuals with severe negative and disorganised symptoms, identifying a subgroup of patients for whom combined interventions would be helpful (Benavides et al. 2018). Thus, it would be useful to recognise factors contributing to low SE in patients with psychosis to facilitate their recovery by positively impacting their quality of life (Barbalat et al. 2022).

Concerning people with 22q11.2DS, low SE has been little investigated (Schneider et al. 2014). Although relationships between cognitive endophenotypes and the development of psychiatric disorders in 22q11DS (Biswas and Furniss 2016) and cognitive impairments with general functioning and social skills (Accinni et al. 2022; Campbell et al. 2015; Frascarelli et al. 2023) have been reported, the relationship of SE with real‐life and social functioning of people with 22q11.2DS has not received attention.

SE can be considered as a multidimensional psychological function, arising from the interaction of different subjective and environmental factors. Hence, it may reflect mutual interactions between personal and subjective psychological abilities and the environment where people with 22q11.2DS are living.

## 2 Aims

We hypothesised that SE levels in a population of people with 22q11.2DS would decrease with increasing severity of psychosis. This study aims to explore the levels of SE in a group of individuals with 22q11.2DS, so as to see whether SE levels vary according to psychotic disorder severity.

Subsequently, we aimed at investigating how levels of SE in people with 22q11.2DS would correlate with patients' real‐life and social functioning and with the severity of psychopathology.

## 3 Methods

The sample consisted of 32 individuals aged between 16 and 66 years, divided into a group of individuals with 22q11.2 microdeletion (DEL, *N* = 22) without psychosis and another group of people with 22q11.2 microdeletion and a psychotic disorder, including schizophrenia, schizophreniform disorder and psychotic disorders not otherwise specified (DEL‐SCZ, *N* = 10). All patients at the time of assessment were on stable medication and in remission from the acute illness phase. Sample recruitment occurred at the Outpatient Clinic for the Treatment of Psychotic Disorders and for Mental Health in 22q11.2DS, from 2014 to 2021. The genetic diagnosis was established through a complete genetic investigation using fluorescent in situ hybridisation (FISH). Individuals with multiple genetic abnormalities or severely impaired cognitive profiles were excluded. Each participant signed free, informed consent. The study adopted the Helsinki 1964 Principles of Human Rights, as amended by the 64th WMA General Assembly, Fortaleza, Ceará, Brazil, October 2013. All data were anonymised. Clinical and demographic data were collected for all participants.

### 3.1 Clinical Evaluation

Each participant underwent psychiatric evaluation using the DSM‐5 criteria. Symptom severity was assessed through the Positive And Negative Syndrome Scale (PANSS) (Kay et al. 1987).

### 3.2 Evaluation of Patients' Real‐Life Functioning

To assess the real‐life functioning of recruited patients, we employed the Specific Levels of Functioning scale (SLoF) (Montemagni et al. 2015; Mucci et al. 2021), a 43‐item multidimensional behavioural interview referring to the past week, administered to the patient's caregiver looking at different domains of patient's functioning and independent life profile: *Self Maintenance*, including *Physical Functioning* and *Personal Care Skills*; *Social Functioning*, involving *Interpersonal Relationships* and *Social Acceptability*; *Community Living Skills*, related to *Activities* and *Work Skills* subscales. The SLoF subscales assess patients' current functioning comprising their observable behaviour without looking at psychopathology and cognitive dysfunction. The potential total scores of the scale range from 43 to 215, with higher total scores (SLoF‐T) indicating better overall patient functioning. In this study, we investigated *Social Functioning* (*Interpersonal Relationships* and *Social Acceptability*) (SLoF‐sf), based on evidence of a tight association between social functioning impairment and psychopathology in schizophrenia (Handest et al. 2023).

### 3.3 Evaluation of Patients' SE

We employed the Self‐Esteem Rating Scale (SERS) (Nugent and Thomas 1993) to evaluate patients' SE. This 40‐item tool is rated on a 7‐point Likert type scale (1 = *Never*, 4 = *Some of the time*, 7 = *Always*) aimed at evaluating different aspects of self‐consideration, like overall self‐worth, social competence, problem‐solving ability, intellectual ability, self‐competence and worth compared with others. Twenty items are scored positively, the other 20 negatively; item scores are summed to produce a total score ranging from −120 to +120, with positive scores indicating higher levels of SE and negative scores lower levels. The SERS has been previously employed in samples with schizophrenia (Galderisi et al. 2018).

All evaluating clinicians were trained in administering the PANSS and the SLoF scales; their interrater reliability has been previously tested and reported (Galderisi et al. 2014).

### 3.4 Statistical Analysis

We employed the Shapiro–Wilk test to verify the normality of data distribution and Levene's test to check for homogeneity of variances. When assumption of normality of the distribution was not met, the Mann–Whitney‐Wilcoxon *U*‐test was employed to investigate between‐groups differences in mean scores. For categorical variables, we used the *χ* ^2^ test; to test mean differences between groups on continuous variable, when normal distribution was met, we used Student *t*‐test. Subsequently, we used Spearman's correlation coefficients (*ρ*) to study the relationships between SERS, PANSS‐T, SLoF‐T and SLoF‐sf (Interpersonal Relationships and Social Acceptability domains) scores. Statistical significance was set at *p* < 0.05 for all analyses. We used the SPSS 25.0 version (Statistical Package for the Social Sciences, IBM Co., Armonk, New York 2017) for all statistical analyses.

## 4 Results

Clinical and demographic characteristics of the recruited sample are shown in Table 1.

**TABLE 1:** Demographical and clinical variables of the recruited sample.

Variables	DEL‐SCZ *N* = 10	DEL *N* = 22	*t*_(*df*)_ or *χ* ^2^	*p*
Mean age ± SD	26.8 ± 8	23.6 ± 7.1	1.356	0.269
Sex (N. female (%))	1 (10%)	10 (45.5%)	3.831	0.050
SERS mean (std err.)	16.5 (9.49)	36.55 (10.03)	−1.232_(30)_	0.228
SLoF‐T mean (std err.)	149.22 (10.94)	181.00 (5.22)	−2.989_(26)_	0.006
SLoF‐sf mean (std err.)	50.11 (3.18)	58.16 (1.92)	−2.277_(26)_	0.031
PANSS‐T mean (std err.)	82.80 (5.14)	53.58 (2.78)	5.482_(27)_	< 0.001

*Note:* Mean scores ± standard deviation (SD) and standard errors (std err.) are represented. For categorical variables, the *χ* ^2^ squared test was employed to assess differences between groups, and statistics have been reported; to test mean differences between groups, the Student's *t*‐test has been performed, and statistics have been reported.

Abbreviations: *χ* ^2^, chi squared test; DEL, 22q11.2 deletion syndrome; DEL‐SCZ, 22q11.2 deletion syndrome and schizophrenia; PANSS tot, PANSS total score; SERS, SERS total score; SLoF‐sf, SLoF social functioning; SLoF‐T, SLoF total score; *t*_(*df*)_, Student's *t‐*test (degrees of freedom).

Homoscedasticity was respected, since Levene's test for all considered variables were not significant in any group (Table 2). In both groups, the considered variables were normally distributed according to the Shapiro–Wilk, except for the PANSS total score in the DEL group; however, all recruited variables showed normal distribution in both groups on the Kolmogorov–Smirnov test. We used the Mann–Whitney *U*‐test to compare DEL and DEL‐SCZ on PANSS‐T and found significant differences (*U* = 16.5; *p* < 0.001), similar to those found with the parametric Student's *t*‐test, whereby the DEL‐SCZ group scored higher on the PANSS‐T compared to the DEL group (*F* = 0.563; *t* = 5.482; *p* < 0.001). DEL and DEL‐SCZ did not significantly differ on the SERS (*F* = 1.39; *t* = −1.232; *p* = 0.228). The DEL group scored higher than DEL‐SCZ on the SLoF‐T (*F* = 1.267; *t* = −2.989; *p* = 0.006) and on the SLoF‐sf (*F* = 0.595; *t* = −2.277; *p* = 0.031). We calculated Cohen's *d* effect size for differences between groups for all considered variables (Table 2).

**TABLE 2:** Levene's test and Cohen's *d* effect sizes for differences between groups in all considered variables.

	Standardised	Estimate of point	Inferior CI (95%)	Superior CI (95%)	*F*	*p*
SERS	42.67	−0.470	−1.223	0.291	1.399	0.246
SLoF‐T	26.28	−1.209	−2.057	−0.341	1.267	0.271
SLoF‐sf	8.73	−0.921	−1.745	−0.082	0.595	0.448
PANSS‐T	13.64	2.142	1.175	3.082	0.563	0.460

Abbreviations: DEL, 22q11.2 deletion syndrome; DEL‐SCZ, 22q11.2 deletion syndrome and schizophrenia; PANSS‐T, PANSS total score; SERS, SERS total score; SLoF‐sf, SLoF social functioning; SLoF‐T, SLoF total score.

**FIGURE 1:**
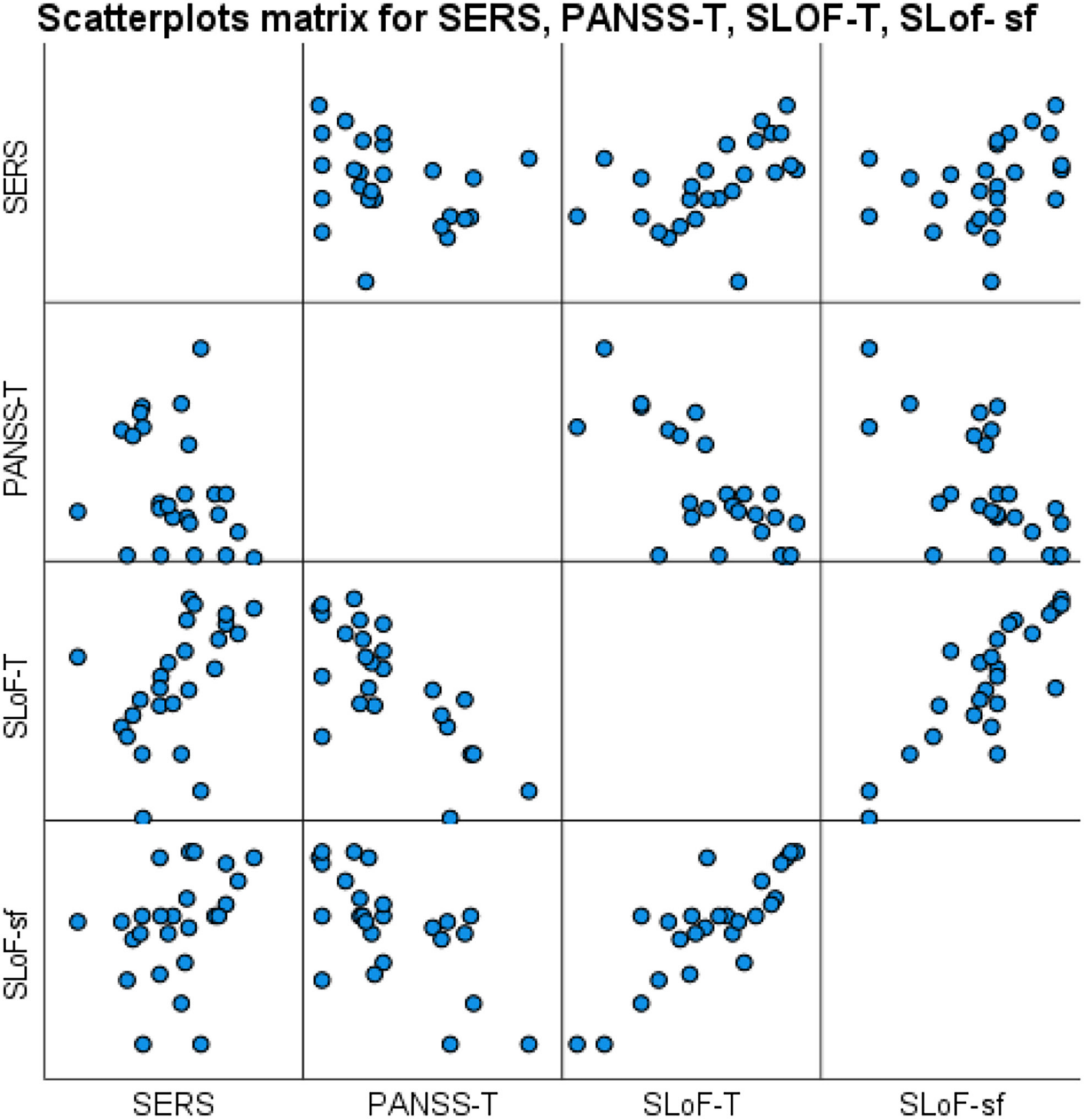
Scatterplots' matrix for considered variables. PANSS‐T = PANSS total score; SERS = SERS total score; SLoF‐sf = SLoF social functioning; SLoF‐T = SLoF total score.

PANSS‐T negatively correlated with SLoF‐T scores (*ρ* = −0.698; *p* < 0.001), with SLoF‐sf (*ρ* = −0.643; *p* < 0.001) and with SERS (*ρ* = −0.391; *p* = 0.036). SERS positively correlated with SLoF‐T (*ρ* = 0.545; *p* = 0.003) but not with SLoF‐sf, that is, Interpersonal Relationships and Social Acceptability (Figure 1).

## 5 Discussion

This cross‐sectional study aimed at preliminarily evaluating whether the presence of a full‐blown psychotic disorder is associated with lower SE levels in patients with 22q11.2DS and to identify correlations between SE, psychopathology and general and social functioning in people with 22q11.2DS. The choice to focus on the social domain derives from the evidence that symptom severity in schizophrenia is tightly associated with impairments in social skills and interpersonal abilities (Accinni et al. 2022; Handest et al. 2023). We found that DEL and DEL‐SCZ groups did not differ on SE, but the latter scored higher on psychopathology and lower on specific levels of functioning and specifically, on social functioning. SE was positively correlated with total levels of functioning and negatively correlated with psychopathological severity.

The lack of differences between DEL and DEL‐SCZ in SE suggests that this psychological dimension is not particularly associated with the presence of full‐blown psychosis. SE is a complex dimension referring to self‐evaluation and involves both cognitive and affective factors (Pelham et al. 1989). We may hypothesise that people carrying the 22q11.2 microdeletion, independently from the presence or absence of psychosis, suffer from a highly penetrant syndrome affecting several different biological systems, in turn impacting their physical functioning. Low functioning could lead to low SE in people with 22q11.2DS, regardless of the presence of psychotic symptoms; we do not know whether both DEL and DEL‐SCZ groups score low on SE since we had not a normal control group. Alternatively, we may hypothesise that the low insight of 22q11.2DS people with negative symptoms after their psychotic onset could have dampened the detrimental effect of reduced functioning on SE. While SE has been extensively investigated in schizophrenia and the psychoses (Bemrose et al. 2021; Ciufolini et al. 2015), it has not been assessed in 22q11.2DS.

Our results corroborate the concept that higher levels of psychopathology in individuals with 22q11.2DS significantly correlate with lower real‐life functioning, particularly in social and interpersonal functioning, and lower SE, as previously reported (Accinni et al. 2022; Calderon‐Mediavilla et al. 2021; Handest et al. 2023).

Subsequently, we found that SE and psychopathology were positively and negatively correlated, respectively, with the general real‐life functioning of individuals with 22q11.2DS. SE scores were inversely correlated with PANSS, which also measures depressive symptoms, leading us to infer that SE is more strongly associated with these symptoms than with psychotic ones. The latter were inversely correlated with social functioning, as previously reported (Accinni et al. 2022; Handest et al. 2023), whereas levels of SE were not. These findings seem to confirm that SE is related to patients' real‐life functioning differently from psychotic symptoms. We may hypothesise that while psychotic symptoms correlate with social functioning of patients with 22q11.2DS, including interpersonal and social abilities, SE (conceived as the self‐evaluation process based on mentalisation abilities) may rather correlate with those aspects of real‐life functioning which are strongly associated to environmental factors and volitional elements: psychosis would impair social functioning of patients who would misinterpret social roles and others' intentions, thus leading to an attribution of subjective meanings to interpersonal interactions that may even result in persecutory experiences (Moore et al. 2006). However, our cross‐sectional design does not allow such speculations.

### 5.1 Limitations

Our preliminary results point to an interaction between different variables and the real‐life and social functioning of individuals at higher genetic risk of developing psychosis. Limitations of the study include small sample size (however, 22q11.2DS is a rare genetic syndrome complicating patient recruitment), the lack of a healthy control group, the absence of covariates and the cross‐sectional nature that prevents the establishment of causality. Future studies could collect longitudinal data and include other assessments, such as suicidal risk, attachment style, resilience, internalised stigma, coping and locus of control, which have not yet been investigated in relation to SE in 22q11.2DS.

## 6 Conclusions

Our preliminary data suggest that SE in 22q11.2DS is not directly associated with a full‐blown psychotic disorder. While psychopathology appears to be directly related to patients' social functioning, levels of SE seem to be linked to other aspects of real‐life functioning, likely those most influenced by environmental factors.

## Author Contributions

All authors viewed and approved the final version of the manuscript: T.A. and A.B. conceived and designed the project, T.A., A.B. and E.C.I. acquired the data, T.A., A.B., G.D.K. and E.C.I. analysed and interpreted the data, T.A. and G.D.K. wrote the paper and M.P. supervised the study.

## Ethics Statement

The study adopted the Principles of Human Rights, as issued by the World Medical Association at the 18th WMA General Assembly, Helsinki, Finland, June 1964, and subsequently amended by the 64th WMA General Assembly, Fortaleza, Brazil, in October 2013, and received approval by the Ethics Committee of the Umberto I Hospital, Rome, protocol. 5341 n.250/19. All patient and other participant data were anonymised.

## Conflicts of Interest

The authors declare no conflicts of interest.

## Data Availability

The data that support the findings of this study are available on request from the corresponding author. The data are not publicly available due to privacy or ethical restrictions.
